# Complications Following Bilateral Salpingectomy by Indication: Population‐Based Cohort Study

**DOI:** 10.1111/1471-0528.70143

**Published:** 2026-01-07

**Authors:** Alexandra Lukey, Helena Abreu do Valle, A. Fuchsia Howard, Celeste Leigh Pearce, David G. Huntsman, Janice S. Kwon, Jessica N. McAlpine, Michael R. Law, Paramdeep Kaur, Rafael Meza, Gillian E. Hanley

**Affiliations:** ^1^ Department of Gynaecology and Obstetrics, Division of Gynaecologic Oncology University of British Columbia Vancouver British Columbia Canada; ^2^ School of Population and Public Health University of British Columbia Vancouver British Columbia Canada; ^3^ Faculty of Applied Sciences University of British Columbia, School of Nursing Vancouver British Columbia Canada; ^4^ University of Michigan Ann Arbor Michigan USA; ^5^ BC Cancer Research Institute University of British Columbia Vancouver British Columbia Canada; ^6^ O'Brien Institute for Public Health University of Calgary Calgary Canada; ^7^ Integrative Oncology Program British Columbia Cancer Research Institute Vancouver British Columbia Canada

**Keywords:** cancer prevention, contraception, salpingectomy, surgical complications, tubal contraception

## Abstract

**Objective:**

Assess the short‐term surgical outcomes of bilateral salpingectomy performed as a standalone procedure, focusing on complication rates by surgical indication and age group.

**Design:**

Retrospective population‐based cohort.

**Setting:**

British Columbia, Canada, from January 1st, 2008, to December 31st, 2022.

**Population:**

7102 people who received bilateral salpingectomies performed without concurrent surgical procedures.

**Methods:**

International Disease Classification codes were used to identify the indication for bilateral salpingectomy. We compared outcomes for salpingectomy performed for prophylactic versus contraceptive indications, as well as across different age groups.

**Main Outcome Measures:**

The primary outcome was a composite measure of complications assessed up from the index surgery to 6 weeks after discharge. We included admission to the intensive care unit, return to the operating room, in‐hospital surgical complications, readmissions, and complications diagnosed during physician visits.

**Results:**

There were 197 complications out of 7102 surgeries for bilateral salpingectomy corresponding to an overall complication rate of 2.8%. Complications occurred in 2.7% of procedures performed for contraception and 4.5% of those performed for prophylaxis, with no statistically significant difference between groups. There were also no significant differences in the adjusted risk ratios for same day discharge, postoperative complications, diagnostic imaging, or prescriptions for NSAIDs or opioids between indication groups. No significant differences in any of the measured outcomes were observed across age groups (< 35, 35–45, and > 45 years).

**Conclusion:**

These results illustrate low complication rates in people undergoing bilateral salpingectomy as a standalone surgical procedure.

## 1 Introduction

Salpingectomy, has historically been used as a standalone procedure for the indications of certain benign gynaecologic diseases, and in emergent cases, such as ectopic pregnancies [1, 2]. After evidence of the aetiology of high grade serous ovarian cancer began to illustrate that the fallopian tube was the tissue of origin for most high grade serous ovarian cancers [3, 4, 5, 6], bilateral salpingectomies (BS) have been added to many other benign gynaecologic surgeries [7]. This approach has been termed ‘opportunistic salpingectomy’ (OS) [8].

With increasing uptake [9, 10], strong evidence of safety for OS [11, 12, 13, 14], and evidence for the effectiveness of OS as a method of ovarian cancer prevention [1, 15], more interest is being paid to how salpingectomy can be used to dramatically decrease the incidence of high grade serous ovarian cancer [16]. Other than OS, salpingectomy could also be offered as a standalone surgery to people at higher‐than‐average risk for ovarian cancer. While people who meet criteria for risk‐reducing bilateral salpingo‐oophorectomy (RRBSO), should still be offered RRBSO [17, 18], individuals whose risk profile puts them at a higher likelihood of developing a serous ovarian cancer may be counselled to consider BS. Previous research has suggested that a person's lifetime risk of developing ovarian cancer can be estimated by combining polygenic risk scores with other known risk and protective factors [19].

While considerable safety data for OS has been generated, it has always compared the risks of including a salpingectomy with an existing surgery, given that OS patients are, by nature, already having another surgery [12, 13, 14, 20]. Given that risk‐reducing salpingectomy (RRS) is not regularly practiced, the closest comparison group is those undergoing BS for contraception, as this is necessarily a stand‐alone salpingectomy and thus the same surgical procedure that would be performed for RRS [13]. However, to understand whether salpingectomy could be safely offered to people at higher‐than‐average lifetime risk for ovarian cancer, we need better data on complication rates for those undergoing only a salpingectomy.

In this study, we use population‐based administrative data to explore short‐term clinical outcomes in all patients who underwent surgery solely for the purpose of fallopian tube removal. We also examined complication rates across two indications for BS without pathology (BS for contraception and BS for cancer prophylaxis (RRS)), and across different age groups of patients.

## 2 Materials and Methods

This is a retrospective population‐based cohort study using administrative data from British Columbia (BC), Canada, between January 1st, 2008, and December 31st, 2022. The datasets include all residents of BC including all BS performed without other procedure codes listed in the same surgical episode for either contraceptive or prophylactic indications. The following data sets from Population Data BC were used in this study and linked using de‐identified study specific patient IDs provided by programmers at PopData BC: the Discharge Abstract Database, the Medical Services Plan data, the BC PharmaNet, the BC Cancer Registry, Vital Statistics, and the Consolidation Files, providing us with surgeries, medical visits, medication dispensations, cancer diagnoses, demographic and income information, respectively. You can find further information regarding these data sets by visiting the PopData project webpage at: https://my.popdata.bc.ca/project_listings/21‐105/collection_approval_dates. Ethical approval was obtained from the University of British Columbia Clinical Research Ethics Board (H21‐01900).

### 2.1 Study Population

We identified all patients who had BS as a single procedure using the Canadian Classification of Health Intervention (CCI) code 1.RF.89.X along with a code indicating it was bilateral and required that no other procedure codes were present (unless the procedure was a return to the operating room (OR), which was included as an outcome) (Table S1). To ensure data accuracy and completeness, we excluded people who were not of female biological sex, who had BS coded more than once, who had a non‐gynaecologic condition as their primary indication for surgery and anyone was a non‐BC resident. Other exclusion criteria were having undergone an emergency surgery, having a cancer diagnosis listed in the hospital admission for the procedure or salpingectomy done during caesarean section or the same hospital stay as a live birth.

### 2.2 Independent Variables

We stratified our cohort into two groups based on their surgical indication for BS using their ICD 10 diagnostic codes: (1) Contraceptive surgery and (2) Prophylactic surgery (Table S2, Benign indications excluded). We used the first coded diagnosis which is the most responsible diagnosis in the hospital data to define these groups. We were further interested in the differences in age at which BS occurred, which may be important to understand the recommendation of timing. Therefore, we also compared outcomes between those who underwent BS under 35, 35–45 and over 45 years of age.

### 2.3 Outcomes

We examined perioperative outcomes including OR time (time from first skin incision until completed skin closure), length of hospital stay, return to the OR, admission to intensive care unit, and surgical complications such as haemorrhage or pelvic organ injury identified through ICD 10 diagnostic codes during the hospital stay (Tables S3 and S4). We also analysed post‐operative outcomes up to six weeks following the hospital discharge including hospital readmissions, physician visits with an ICD 9 diagnostic code likely related to a surgical complication (Table S5) ultrasound imaging or lab tests ordered and the likelihood of filling a prescription for an antibiotic or a prescription‐strength analgesic identified using Anatomical Therapeutic Classification Codes (Table S6). Large differences in the rates of filling these prescriptions could indicate differences in the likelihood of infection, and pain between the prophylactic and contraceptive groups. For each medication category (antibiotic, NSAID, or opioid), we report any prescription filled after the hospital discharge, up to six weeks. We also report the number of days dispensed, using the ‘days dispensed’ variable from the PharmaNet database. By law, the BC PharmaNet records all prescription medications that are dispensed outside of hospital settings. Our primary outcome was a composite of complications that included any of the following: admission to intensive care unit, return to the OR, surgical complications, readmissions, and complications diagnosed during physician visits. This relatively wide criteria allowed us to include any complications that cause harm even if the effects were not lasting. We also show rates for each complication separately, and report minor compared to major complications, as well as including data on additional outcomes that may be of interest but are not included in our primary outcome (i.e., NSAID, antibiotic and opioid use).

### 2.4 Statistical Analysis

We compared the characteristics of the study groups using standardised mean differences (SMD) and if SMD was greater than 0.1 it was considered a meaningful difference between the groups. We chose to use SMD, as with large sample sizes, *p*‐values will almost always be small [21] We used log‐binomial regression models to estimate risk ratios for binary outcomes. Due to the low number of events, we grouped these events as a composite outcome for our model: return to the OR, admission to the intensive care unit, surgical complications, hospital readmissions and complications diagnosed during physician visits. Further, where cells had five or fewer cases, only approximate numbers are presented due to privacy agreements with the data stewards. Baseline characteristics were tested in our models for theoretical viability and the change in estimate criterion for confounder adjustment. In models where age was the independent variable, indication for BS was not included in the models as this was determined to be on the causal pathway. In cases where adjustment variables had missing data (income and health authority), we first created a separate ‘missing’ category for modelling. We then performed sensitivity analyses in which these variables were imputed using hot‐deck imputation [22] Statistical analyses were performed with R software version 4.0.5.

## 3 Results

We included 7102 BSs performed without additional surgeries, the majority of which were performed for the indication of contraception (6970, 98.1%) (Figure S1). The mean age of individuals who underwent BS was 35.9 years (standard deviation (SD), 5.9) (Table 1). Those who had BS for prophylactic indications were older than those who had BS for contraception, with a mean age of 43.7 (SD, 8.6) compared to 35.7 (SD, 5.69) years for contraception patients. Individuals who received BS for contraception also had fewer previous gynaecologic surgeries.

**TABLE 1:** Characteristics of people who had BS as a single procedure by surgical indication in British Columbia between 2008 and 2022.

	Overall (*N* = 7102)	Contraception (*N* = 6970)	Prophylactic (*N* = 132)	SMD^a^
**Age in years**, mean (SD), Range	35.9 (5.9) (19.7–68.8)	35.7 (5.7) (19.7–57.2)	43.7 (8.6) (24.4–68.8)	1.09
**Age category**				
< 35	3079 (43.4)	3057 (43.9)	22 (16.7)	1.02
35–45	3779 (53.2)	3717 (53.3)	62 (47.0)	
> 45	244 (3.4)	196 (2.8)	48 (36.4)	
**Year of surgery, median** (IQR)	2017 (5)	2017 (5)	2016 (6)	0.20
**Route of surgery (%)**				
Laparoscopic	7023 (99.0)	6892 (98.9)	131 (99.2)	0.05
Abdominal	~75	~70	≤ 5^b^	
Vaginal	≤ 5^b^	≤ 5^b^	0 (0.0)	
**Previous gynecologic surgery**, median (IQR)	0 (1.0)	0 (1.0)	0 (1.0)	0.06
**Previous gynecologic surgery number (%)**				
0	5147 (72.5)	5050 (72.5)	97 (73.5)	0.12
1	977 (13.8)	962 (13.8)	15 (11.4)	
2	609 (8.6)	599 (8.6)	10 (7.6)	
≥ 3	369 (5.2)	359 (5.2)	10 (7.6)	
**Income quintile (%)**				
1	1501 (21.1)	1495 (21.4)	6 (4.5)	0.61
2	1407 (19.8)	1379 (19.8)	28 (21.2)	
3	1312 (18.5)	1289 (18.5)	23 (17.4)	
4	1255 (17.7)	1231 (17.7)	24 (18.2)	
5	982 (13.8)	943 (13.5)	39 (29.5)	
Missing	645 (9.1)	633 (9.1)	12 (9.1)	
**Health authority area (%)**				
Fraser	2699 (38.0)	2657 (38.1)	42 (31.8)	0.51
Interior	1802 (25.4)	1780 (25.5)	22 (16.7)	
Northern	869 (12.2)	862 (12.4)	7 (5.3)	
Vancouver Coastal	654 (9.2)	630 (9.0)	24 (18.2)	
Vancouver Island	1060 (14.9)	1024 (14.7)	~35	
Missing	~20	17 (0.2)	≤ 5^b^	
**Number of live births**				
Median (IQR)	2 (1.0)	2 (1.0)	2 (0)	0.59
Missing (%)	1356 (18.2)	1164 (16.7)	47 (35.6)	
**Time since last pregnancy in years**, mean (SD)	3.6 (7.4)	3.5 (7.3)	7.5 (11.6)	0.64

^a^ SMD > 0.1 means significant differences.

^b^ Cannot publish < 5 cell sizes because of privacy agreements with data stewards.

### 3.1 Complications

Of the 7102 people who underwent BS, the rate of any complication (our primary outcome) was 2.8%, representing an absolute risk of 28 per 1000 surgeries (Table 2). No fatal complications were observed and there were fewer than five ICU admissions or returns to the OR. We further divided the complications into major complications (admission to intensive care unit, return to the OR, in hospital surgical complications, readmissions related to surgical complications) and minor complications (complications diagnosed during outpatient physician visits not resulting in readmission). The rate of major complications was 0.7% and the rate of minor complications was 1.9%. The most frequent codes related to outpatient complications were related to inflammatory disease and other non‐specified complications related to medical care (Table S7). There were too few major complications to further disaggregate these codes. Further, there were approximately ~115 physician visits in the 6 weeks of follow up for infection and ~45 for other complications.

**TABLE 2:** Frequency of complications overall and by indication for BS in British Columbia between 2008 and 2022.

	Overall (*N* = 7102)	Contraception (*N* = 6970)	Prophylactic (*N* = 132)	SMD
**Length of hospital stay in hours, median (IQR)**	5 (2)	5 (2)	6 (2)	0.22
**OR time in minutes, median (IQR)**	56 (18)	56.0 (18)	59 (15.5)	0.30
**Same day discharge (%)**	7044 (99.2)	6918 (99.3)	126 (95.5)	0.24
**Any complication** ^**a**^ **(%)**	197 (2.8)	191 (2.7)	6 (4.5)	0.10
**Major complication (%)** ^**b**^	50 (0.7)	~45	≤ 5^d^	0.08
**Minor complication (%)** ^**c**^	150 (1.9)	~145	≤ 5^d^	0.06
**Admission to the intensive care unit (%)**	≤ 5^d^	≤ 5^d^	≤ 5^d^	0.02
**OR return (%)**	≤ 5^d^	≤ 5^d^	≤ 5^d^	0.02
**In hospital complication (%)**	~15	15 (0.2)	≤ 5^d^	0.08
**Readmission (%)**	~80	77 (1.1)	≤ 5^d^	0.04
**Readmission likely due to surgical complication (%)**	~35	32 (0.5)	≤ 5^d^	0.04
**Number of physician visit, mean (SD)**	1.1 (3.1)	1.1 (3.1)	1.6 (3.6)	0.12
**Physician visit – infection (%)**	~115	109 (1.6)	≤ 5^d^	< 0.01
**Physician visit – other complication (%)**	~45	44 (0.6)	≤ 5^d^	0.09
**Diagnostic test (%)**	847 (11.4)	771 (11.1)	18 (13.6)	0.12
**X ray ordered (%)**	232 (3.3)	225 (3.2)	7 (5.3)	0.10
**Ultrasound ordered (%)**	~170	166 (2.4)	≤ 5^d^	0.04
**Lab test ordered (%)**	710 (10.0)	693 (9.9)	17 (12.9)	0.09
**Antibiotics (%)**	668 (9.4)	654 (9.4)	14 (10.6)	0.04
Number of antibiotics days dispensed amongst users, mean (SD)	9.8 (9.1)	9.8 (9.1)	10.9 (9.0)	0.13
**NSAID (%)**	2043 (28.8)	2002 (28.7)	41 (31.1)	0.05
Number of NSAIDS days dispensed amongst users, mean (SD)	10.0 (7.4)	10.0 (7.2)	10.8 (14.8)	0.07
**Opioids (%)**	3598 (50.7)	3532 (50.7)	66 (50.0)	0.01
Number of days Opioids dispensed, mean (SD)	4.5 (5.5)	4.5 (5.5)	3.3 (1.8)	0.29

*Note:* SMD > 0.1 indicates a clinically meaningful difference.

^a^ Admission to intensive care unit, return to the OR, in hospital surgical complications, readmissions, and complications diagnosed during physician visits.

^b^ Admission to intensive care unit, return to the OR, in hospital surgical complications and readmissions.

^c^ Complications diagnosed during physician visits.

^d^ Cannot publish numbers between 1 and 4 because of privacy agreements with data stewards.

With respect to differences across the surgical indication groups, BS performed for contraception had the lowest complication rate at 2.7% and BS for prophylaxis had a complication rate of 4.5% (SMD 0.10). The readmission rate for any reason up to six‐weeks post‐discharge for BS performed for contraception was 1.1%, or 11 per 1000 surgeries. The number of readmissions for any reason for the prophylactic indication was too small to share due to privacy considerations (number between 1 and 5). After controlling for confounders, there was no statistically significant difference in the relative risk between BS performed for prophylactic indications and BS performed for contraception for same day discharge, complications, post‐operative diagnostic tests (laboratory, X‐ray and ultrasound) or prescriptions of NSAIDs or opioids. (Table 3).

**TABLE 3:** Unadjusted and adjusted risk ratio for the outcomes by indication of BS in British Columbia between 2008 and 2022.

	Contraception (*N* = 6970)		Prophylactic (*N* = 132)		
	Events	Reference	Events	Crude risk ratio (95% CI)	Adjusted risk ratio (95% CI)
Same day discharge	6918	1.00	126	0.96 (0.93–1.00)	0.96 (0.93–1.00)
Any complication^a^	191	1.00	6	1.66 (0.75–3.67)	1.77 (0.76–4.14)
Diagnostic test^b^	771	1.00	18	1.23 (0.80–1.90)	1.23 (0.79–1.92)
Prescription analgesic use: NSAIDS	2002	1.00	41	1.08 (0.84–1.40)	1.11 (0.86–1.45)
Prescription analgesic use: Opioids use	3532	1.00	66	0.99 (0.83–1.17)	0.99 (0.83–1.18)

*Note:* Models were adjusted by age, income, health authority and number of previous gynecologic surgeries.

^a^ Admission to intensive care unit, return to the OR, surgical complications, readmissions, and complications diagnosed during physician visits.

^b^ Diagnostic test includes X‐ray, ultrasound or blood test.

### 3.2 Complications by Age

Most individuals in our cohort were younger than 45 years old (96.5%) (Table S8). Notably, the indication for BS was contraception in 99.3% of people under 35 and 98.4% of people ages 35–45. When examining our primary outcome of any complication, the rates were similar between age groups: under 35 (3.0%), 35–45 (2.6%), over 45 (2.5%) (SMD, 0.02). After adjustment for confounders, there were also no significant differences in the relative risk of same day discharge, complications, diagnostic tests ordered, or NSAID or opioid use comparing the older age groups to those under 35 (Table 4).

**TABLE 4:** Unadjusted and adjusted risk ratios for the outcomes by age group when BS was received in British Columbia between 2008 and 2022.

< 35 years (*N* = 3147)			≥ 35 and ≤ 45 years (*N* = 3910)			> 45 years (*N* = 377)		
	Events	Reference	Events	Crude odds ratio (95% CI)	Adjusted odds ratio (95% CI)	Events	Crude odds ratio (95% CI)	Adjusted odds ratio (95% CI)
Same day discharge	3057	1.00	3747	1.00 (0.99–1.00)	1.00 (0.99–1.00)	240	0.99 (0.97–1.01)	1.00 (0.90–1.01)
Any complication^a^	91	1.00	100	0.90 (0.68–1.18)	0.88 (0.66–1.17)	6	0.83 (0.32–1.76)	0.77 (0.34–1.75)
Diagnostic test^b^	336	1.00	425	1.03 (0.90–1.18)	1.03 (0.90–1.18)	28	1.05 (0.73–1.51)	1.04 (0.72–1.50)
Prescription analgesic use: NSAIDS	868	1.00	1108	1.04 (0.96–1.12)	1.05 (0.98–1.13)	67	0.97 (0.79–1.20)	1.02 (0.98–1.13)
Prescription analgesic use: Opioids use	1584	1.00	1874	1.14 (0.96–1.12)	1.05 (0.98–1.13)	140	0.97 (0.79–1.20)	1.02 (0.98–1.13)

*Note:* Models were adjusted by year of surgery, income, health authority and number of previous gynecologic surgery.

^a^ Admission to intensive care unit, return to the OR, surgical complications, readmissions, and complications diagnosed during physician visits.

^b^ Diagnostic test includes, X‐ray, ultrasound or blood test.

## 4 Discussion

### 4.1 Main Findings

In this cohort of 7102 individuals who underwent BS without any additional concomitant procedures for either contraceptive or prophylactic indications between 2008 and 2022, the overall complication rate was 2.8%, most of which were minor. There were fewer than five admissions to the ICU or returns to the OR. Overall, these findings suggest that BS as a stand‐alone surgery for patients requesting ovarian cancer prevention has a comparable rate of complications to BS done for contraception. After adjustment, there was no significant difference in complication rates between BS for a prophylactic indication or contraception indication. While rates of prophylactic BS were low in this cohort, our results suggest that complication rates would likely lie somewhere between the contraception group and the prophylaxis groups if RRS were offered to people at higher‐than‐average lifetime risk for ovarian cancer.

### 4.2 Strengths and Limitations

In measuring the complication rate of RRS, we benefit from a dataset collected in an area where salpingectomy is already being used explicitly for prophylactic purposes. We also benefit from being in a region where there has been considerable uptake of salpingectomy for permanent contraception to reduce ovarian cancer risk [9] This allowed us to make comparisons to salpingectomy performed for contraceptive purposes. Furthermore, there is a high degree of certainty regarding our conclusions about serious complications as it is unlikely that we underestimated serious complications, such as events leading to ICU admission or return to the operating room, as these tend to be coded accurately, given their severity.

There are also several important limitations to this study. Firstly, the generalisability of these findings must be interpreted with caution. British Columbia is unique due to its relatively long history of uptake of OS [23] Even so, a limited number of cases were performed for ‘prophylactic’ indications. However, qualitative work on the same topic suggests that a proportion of salpingectomies billed as contraceptive procedures may have been influenced by a patient's desire for cancer prevention, as nuanced discussions between physicians and patients often address multiple indications [24] therefore, fully differentiating intent is limited. While it is a limitation that we rely on a small number of procedures performed with the indication of prophylaxis and thus draw many safety conclusions from procedures done for contraceptive indications, salpingectomy for contraception is an identical procedure to the salpingectomy for prophylaxis, and it is simply the patient population that may ultimately differ slightly. Thus, this does limit the certainty of our conclusions, but extrapolating from these data is not inappropriate.

Further, our choice of what complications to include in our composite outcome was subject to data availability. The list of conditions included affected our rates of complications; however, we ensured that major complications, including hospital readmissions, return to the OR, and pelvic organ injury were captured in the composite. Finally, the administrative databases that we used do not include some potentially important confounding variables such as education.

### 4.3 Interpretation

Our study is comparable to previous studies reporting BS complication rates but is the first population‐based study to focus solely on complication rates in people who have undergone salpingectomy without any other concurrent procedures in the same surgical episode in an attempt to understand the absolute risks of RRS. In the United States, the postoperative complication rate was 4.3% in a cohort of 10 697 individuals who underwent hysterectomy with BS [11]. In another British Columbia cohort which measured outcomes up to 2014, individuals who received BS for contraception had a mean of 2.3 physician visits in the two weeks following surgery [13]. This was higher than what we observed in our cohort, which had a mean of 1.13 physician visits. Another smaller study of 180 BS reported an intraoperative or postoperative complication rate of 2.8% [25]. Overall, the complication rates observed in our study and previous research on BS are lower than the total adverse event rate for any gynaecologic surgery, which has a median complication rate of 6.2% (range: 5.7% to 6.7%) [26]. However, as RRS does not include another indication for surgery and is preventative in nature, the acceptable threshold for complication rates would likely be lower than acceptable thresholds for curative gynaecologic surgeries.

The risk of complications in non‐cardiac surgeries increases significantly above the age of 65–70 [27, 28]. However, RRS would be recommended much earlier than this age, given that the average age of diagnosis for high grade serous carcinoma is 64 [29]. Previous research examining complication rates by age for female tubal contraception found decreased odds of complications for individuals under 30 compared to those over 40, but no significant difference between those under 30 and those between 30 and 40 [30]. We found no significant relationship between age and complications in this cohort. Given the high likelihood that serous tubal intraepithelial carcinoma (STIC) lesions will progress to high grade serous ovarian cancers [31], and the clear relationship between age and development of a STIC lesion [6, 32], removal of fallopian tubes before STIC lesions (or precursor p53 lesions) can develop would be optimal. Thus, to achieve the greatest risk reduction for ovarian cancer while keeping the likelihood of complications low, we would ideally intervene as soon as the patient is ready for irreversible contraception. This would require that counselling is done very carefully given that permanent contraception regret is an important complication that we were unable to measure and is higher in people of younger ages [33].

There is currently no formal recommendation for individuals who do not have a pathogenic variant that increases their risk for ovarian cancer to undergo any prophylactic surgery for ovarian cancer prevention. However, our data indicates that this is already occurring at low rates given that we found a small cohort of patients who underwent BS for indications that were coded as ‘prophylactic’. It is also possible that some of these prophylactic surgeries are occurring in people with pathogenic variants as a way to delay oophorectomy and premature surgical menopause. However, prior to moving ahead with targeted RRS for people with no pathogenic variants who are at higher‐than‐average lifetime risk for ovarian cancer, we need to understand the baseline complication rates, which will help us understand the appropriate lifetime risk at which offering RRS is warranted. While this study is an important first step, future studies with larger, prospective datasets are needed to better understand the role of RRS in cancer prevention for the general population. Currently, several clinical trials are underway to evaluate the safety and efficacy of RRS or RRS with delayed oophorectomy in high‐risk populations, such as those with *BRCA1/2* genetic variants to avoid the adverse effects of oophorectomy [34, 35, 36, 37, 38, 39, 40]. These trials will provide valuable insights into the surgical risks associated with salpingectomy for cancer prevention, however, these populations may differ from those in the general population.

## 5 Conclusions

This paper provides an estimate of the underlying complication rates in patients undergoing BS as a standalone surgery and confirms that this rate is low and that complications are largely minor in the six weeks post discharge, with patients usually discharged on the same day as their surgery. While our estimates are still mainly drawn from those receiving BS for contraception indications, this is the same surgical procedure that would be performed for risk‐reducing salpingectomy. There are also currently no guidelines that recommend BS as a stand‐alone surgery for those who wish to reduce their risk of ovarian cancer without another indication for surgery. In summary, our study provides important data to use while considering the expansion to RRS for individuals at higher‐than‐average lifetime risk for ovarian cancer.

## Author Contributions

**Alexandra Lukey:** conceptualization, data curation, formal analysis, funding acquisition, investigation, methodology, writing – original draft, writing – review and editing. **Helena Abreu do Valle:** conceptualization, data curation, formal analysis, writing – review and editing. **A. Fuchsia Howard:** supervision, writing – review and editing. **Celeste Leigh Pearce:** funding acquisition, writing – review and editing. **David G. Huntsman:** funding acquisition, supervision, writing – review and editing. **Janice S. Kwon:** supervision, writing – review and editing. **Jessica N. McAlpine:** supervision, writing – review and editing. **Michael R. Law:** conceptualization, funding acquisition, supervision, writing – review and editing. **Paramdeep Kaur:** data curation, formal analysis, writing – review and editing. **Rafael Meza:** supervision, writing – review and editing. **Gillian E. Hanley:** conceptualization, funding acquisition, investigation, methodology, supervision, writing – review and editing.

## Funding

This work was supported through funding from the Canadian Institutes of Health Research and the Vancouver General Hospital and University of British Columbia Hospital Foundation.

## Ethics Statement

Ethical approval was obtained from the University of British Columbia Clinical Research Ethics Board (H21‐01900).

## Conflicts of Interest

The authors declare no conflicts of interest.

## Supporting information


**Data S1:** bjo70143‐sup‐0001‐Supinfo.docx.

## Data Availability

The data that support the findings of this study are available from Population Data BC. Restrictions apply to the availability of these data, which were used under licence for this study. Data are available from https://my.popdata.bc.ca/project_listings/21‐105/collection_approval_dates with the permission of Population Data BC. Data Sharing: Access to data provided by the Data Stewards is subject to approval but can be requested for research projects through the Data Stewards or their designated service providers. All inferences, opinions, and conclusions drawn in this publication are those of the authors and do not reflect the opinions or policies of the Data Stewards.
