# Supplementary material for: Complications Following Bilateral Salpingectomy by Indication: Population‐Based Cohort Study
Source: BJOG. 2026 Jan 7;133(5):1056–64. doi: 10.1111/1471-0528.70143 (PMC12972845; doi:10.1111/1471-0528.70143)
Supplement: Supplementary file 1 — Data S1: bjo70143‐sup‐0001‐Supinfo.docx. [file BJO-133-1056-s001.docx]

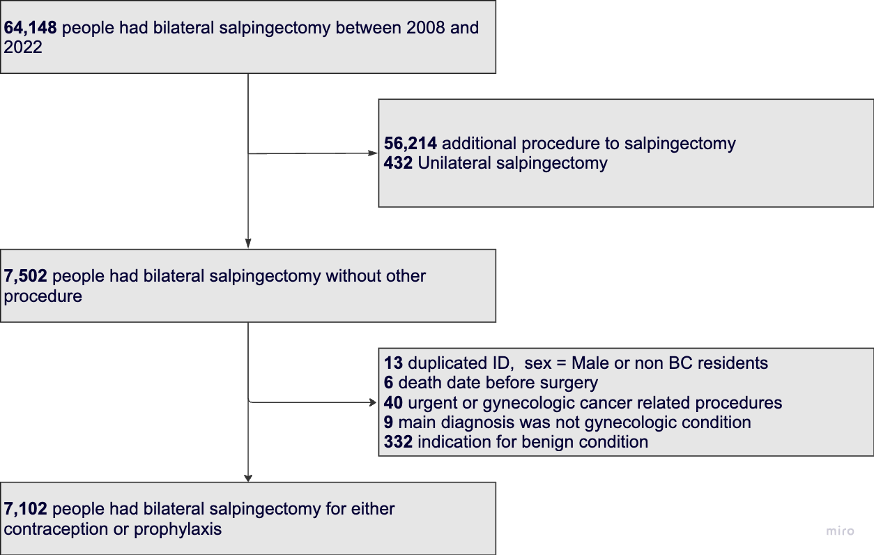
Supplemental Figure 1: Cohort Flowchart

Supplemental Table 1: Canadian Classification of Health Interventions (CCI) surgical procedure codes

| **CCI code** | **Description** |
| --- | --- |
| 1.RB.52 | Drainage, ovary |
| 1.RB.59.^^ | Destruction, ovary |
| 1.RB.74.^^ | Fixation, ovary |
| 1.RB.80 | Repair, ovary |
| 1.RB.87 | Excision partial, ovary |
| 1.RB.89 | Excision total, ovary |
| 1.RD.52 | Drainage, ovary with fallopian tube |
| 1.RD.72 | Release, ovary with fallopian tube |
| 1.RD.89 | Excision total, ovary with fallopian tube |
| 1.RF.52 | Drainage, fallopian tube |
| 1.RF.72 | Release, fallopian tube |
| 1.RF.74.^^ | Fixation, fallopian tube |
| 1.RF.80 | Repair, fallopian tube |
| 1.RF.87 | Excision partial, fallopian tube |
| 1.RF.89 | DATA ngoophorectomy |
| 1.RM.52.^^ | Drainage, uterus and surrounding structures |
| 1.RM.59 | Destruction, uterus and surrounding structures |
| 1.RM.72 | Release, uterus and surrounding structures |
| 1.RM.74.^^ | Fixation, uterus and surrounding structures |
| 1.RM.80 (except for 1.RM.80.BA) | Repair, uterus and surrounding structures |
| 1.RM.87 (except for 1.RM.87.BA) | Excision, partial uterus and surrounding structures |
| 1.RM.89 | Hysterectomy |
| 5.CA.93 | Surgical removal of extrauterine pregnancy |
| 2.RM.70 | Inspection, uterus and surrounding structures |
| 2.OT.70 | Inspection, uterus and surrounding structures |
| 2.RM.71 | Biopsy of uterus and surrounding structures, biopsy of abdominal cavity |
| 2.OT.71 | Biopsy of uterus and surrounding structures, biopsy of abdominal cavity |
| 1.OT.72 | Adhesiolysis, abdominal |
| 5.MD.60 | Cesarean section delivery |
| 1.RB.72 | Manual rupture and drainage of ovarian cyst |
| 1.RB.89.X | Excision total, ovary |
| 1.BF.59 | Uterine nerve ablation, uterosacral nerve ablation |

Supplemental Table 2: Stratification of cohort by surgery indication using ICD 10 diagnostic for main diagnosis for the procedure.

| **Indication group** | **ICD 10 Code** | **Detail** |
| --- | --- | --- |
| Contraception | Z30 | Contraceptive management |
| Prophylactic surgery | Z29 | Need for other prophylactic measures |
|  | Z40 | Prophylactic surgery |
| Benign gynecologic diseases | A18.1 | Tuberculosis of genitourinary system |
|  | A41.9 | Sepsis, unspecified |
|  | D07.3 | Carcinoma in situ of other and unspecified female genital organs |
|  | D25 | Leiomyoma of uterus |
|  | D27 | Benign neoplasm of ovary |
|  | D28.2 | Benign neoplasm of uterine tubes and ligaments |
|  | E28 | Ovarian dysfunction |
|  | K66 | Other disorders of peritoneum |
|  | N70 | Salpingitis and oophoritis |
|  | N80 | Endometriosis |
|  | N83 | Noninflammatory disorders of ovary, fallopian tube and broad ligament |
|  | N92 | Excessive, frequent and irregular menstruation |
|  | N93 | Other abnormal uterine and vaginal bleeding |
|  | N94 | Pain and other conditions associated with female genital organs and menstrual cycle |
|  | N97 | Female infertility |
|  | Q50 | Congenital malformations of ovaries, fallopian tubes and broad ligaments |
|  | R10 | Abdominal and pelvic pain |
|  | R19 | Other symptoms and signs involving the digestive system and abdomen |
|  | R52 | Pain, not elsewhere classified |
|  | R93 | Abnormal findings on diagnostic imaging of other specified body structures |
|  | T83 | Complications of genitourinary prosthetic devices, implants and grafts |
|  | Z01.4 | Gynaecological examination (general)(routine) |
|  | Z03 | Medical observation and evaluation for suspected diseases and conditions, ruled out |
|  | Z12 | Special screening examination for neoplasms |
|  | Z31 | Procreative management |
|  | Z39.0 | Postpartum care and examination |

Supplemental Table 3: ICD 10 codes used to identify complication on hospital files

| **ICD 10 Code** | **Detail** |
| --- | --- |
| R50 | Fever of other and unknown origin |
| T81 | Complications of procedures, not elsewhere classified |
| T83 | Complications of genitourinary prosthetic devices, implants and grafts |
| T85 | Complications of other internal prosthetic devices, implants and grafts |
| Y40 - Y59 | Drugs, medicaments and biological substances causing adverse effects in therapeutic use |
| Y60 - Y69 | Misadventures to patients during surgical and medical care |
| Y70-Y82 | Medical devices associated with adverse incidents in diagnostic and therapeutic use |
| Y83-Y84 | Surgical and other medical procedures as the cause of abnormal reaction of the patient, or of later complication, without mention of  misadventure at the time of the procedure |
| S36 | Injury of intra-abdominal organs |
| S37 | Injury of urinary and pelvic organs |

Supplemental Table 4: Detail on diagnoses not considered as surgical complication (ICD-10 Y83 - Surgical operation as cause of abnormal reaction – was not present with these codes).

| **ICD 10** | **Detail** |
| --- | --- |
| A09 | Other gastroenteritis and colitis of infectious and unspecified origin |
| B07 | Viral warts |
| C18 | Malignant neoplasm of colon |
| D06 | Carcinoma in situ of cervix uteri |
| D24 | Benign neoplasm of breast |
| D30 | Benign neoplasm of urinary organs |
| F10 | Mental and behavioural disorders due to use of alcohol |
| F19 | Mental and behavioural disorders due to multiple drug use and use of other psychoactive substances |
| F20 | Schizophrenia |
| F33 | Recurrent depressive disorder |
| F41 | Other anxiety disorders |
| F43 | Reaction to severe stress, and adjustment disorders |
| F60 | Specific personality disorders |
| G56 | Mononeuropathies of upper limb |
| H26 | Cataract |
| H50 | Strabismus |
| J33 | Nasal polyp |
| J34 | Other disorders of nose and nasal sinuses |
| K02 | Dental caries |
| K20 | Oesophagitis |
| K22 | Other diseases of oesophagus |
| K29 | Gastritis and duodenitis |
| K35 | Acute appendicitis |
| K41 | Femoral hernia |
| K44 | Diaphragmatic hernia |
| K57 | Diverticular disease of intestine |
| K58 | Irritable bowel syndrome |
| K80 | Cholelithiasis |
| M24 | Other specific joint derangements |
| N18 | Chronic kidney disease |
| N393 | Stress incontinence |
| N75 | Diseases of Bartholin's gland |
| O993 | Mental disorders and diseases of the nervous system  complicating pregnancy, childbirth and the puerperium |
| R040 | Epistaxis |
| S64 | Injury of nerves at wrist and hand level |
| S82 | Fracture of lower leg |
| Z12.1 | Special screening examination for neoplasm of intestinal tract |
| Z30.5 | Surveillance of (intrauterine) contraceptive device |
| Z42 | Follow-up care involving plastic surgery |
| Z50.1 | Other physical therapy |
| Z51.2 | Other chemotherapy |

Supplemental Table 5: ICD 9 codes used to identify complication on physician visits files

| **Infection** | |
| --- | --- |
| 038 | Septicemia |
| 567 | Peritonitis and retroperitoneal infections |
| 560.8 | Other specified intestinal obstruction |
| 595.0 | Acute cystitis |
| 614 | Inflammatory disease of ovary fallopian tube pelvic cellular tissue and peritoneum |
| 615 | Inflammatory diseases of uterus except cervix |
| 616 | Inflammatory disease of cervix vagina and vulva |
| 780.6 | Fever and other physiologic disturbances of temperature regulation |
| 998.5 | Postoperative infection not elsewhere classified |
| **Other msp complications** | |
| 996 | Complications peculiar to certain specified procedures |
| 997 | Complications affecting specified body system not elsewhere classified |
| 998* | Other complications of procedures not elsewhere classified |
| 999 | Complications of medical care not elsewhere classified |

*except 9985 that was considered infection

Supplemental Table 6: Anatomical Therapeutic Classification Codes

| **ATC code** | **Detail** |
| --- | --- |
| J01 | Antibiotics |
| M01 | Nonsteroidal anti-inflammatory analgesics (NSAIDs) |
| N02A, N02B | Opiate agonist analgesics |

Supplemental Table 7: Five most frequent out of hospital complications by ICD code

| Code | Description | N |
| --- | --- | --- |
| 616 | Inflammatory disease of cervix vagina and vulva | 38 |
| 614 | Inflammatory disease of ovary fallopian tube pelvic cellular tissue and peritoneum | 23 |
| 999 | Complications of medical care not elsewhere classified | 22 |
| 595.0 | Acute cystitis | 12 |
| 998 | Other complications of procedures not elsewhere classified | 12 |

Supplemental Table 8: Characteristics of people who had BS as a single procedure by age category in British Columbia between 2008-2022

|  | **Overall**  **(N = 7102)** | **< 35 years**  **(N = 3079)** | **35-45 (N = 3779)** | **> 45 years**  **(N = 244)** | **SMD** |
| --- | --- | --- | --- | --- | --- |
| **Age in years,** mean (SD) | 35.9 (5.9) | 30.5 (3.2) | 39.4 (2.9) | 49.1 (3.6) | 3.83 |
| **Year of surgery,** median (IQR) | 2017 (5) | 2017 (6) | 2017 (5) | 2016 (5) | 0.24 |
| **Route of surgery (%)** |  |  |  |  |  |
| Laparoscopic | 7023 (98.9) | 3039 ( 98.7) | 3744 ( 99.1) | 240 ( 98.4) | 0.06 |
| Abdominal | ~75 | ~40 | ~30 | <5 |  |
| Vaginal | <5 | <5 | <5 | <5 |  |
| **Main diagnosis Contraception (%)** | 132 ( 1.9) | 22 ( 0.7) | 62 ( 1.6) | 48 ( 19.7) | 0.45 |
| **Previous gynecologic surgery,** mean (SD) | 0.5 (1.0) | 0.4 (0.9) | 0.6 (1.1) | 0.7 (1.3) | 0.21 |
| **Previous gynecologic surgery number (%)** |  |  |  |  |  |
| 0 | 5147 (72.5) | 2349 ( 76.3) | 2642 ( 69.9) | 156 ( 63.9) | 0.21 |
| 1 | 977 (13.8) | 397 ( 12.9) | 543 ( 14.4) | 37 ( 15.2) |  |
| 2 | 609 ( 8.6) | 224 ( 7.3) | 355 ( 9.4) | 30 ( 12.3) |  |
| >= 3 | 369 ( 5.2) | 109 ( 3.5) | 239 ( 6.3) | 21 ( 8.6) |  |
| **Income quintile (%)** |  |  |  |  |  |
| 1 | 1501 (21.1) | 743 ( 24.1) | 722 ( 19.1) | 36 ( 14.8) | 0.28 |
| 2 | 1407 (19.8) | 629 ( 20.4) | 726 ( 19.2) | 52 ( 21.3) |  |
| 3 | 1312 (18.5) | 561 ( 18.2) | 710 ( 18.8) | 41 ( 16.8) |  |
| 4 | 1255 (17.7) | 488 ( 15.8) | 730 ( 19.3) | 37 ( 15.2) |  |
| 5 | 982 (13.8) | 374 ( 12.1) | 547 ( 14.5) | 61 ( 25.0) |  |
| Missing | 645 ( 9.1) | 284 ( 9.2) | 344 ( 9.1) | 17 ( 7.0) |  |
| **Health authority area (%)** |  |  |  |  |  |
| Fraser | 2699 (38.0) | 1067 ( 34.7) | 1550 ( 41.0) | 82 ( 33.6) | 0.33 |
| Interior | 1802 (25.4) | 881 ( 28.6) | 860 ( 22.8) | 61 ( 25.0) |  |
| Northern | 869 (12.2) | 460 ( 14.9) | 395 ( 10.5) | 14 ( 5.7) |  |
| Vancouver Coastal | 654 ( 9.2) | 199 ( 6.5) | 421 ( 11.1) | 34 ( 13.9) |  |
| Vancouver Island | 1060 (14.9) | 462 ( 15.0) | 545 ( 14.4) | 53 ( 21.7) |  |
| Missing | ~20 | 10 ( 0.3) | 8 ( 0.2) | <5 |  |
| **Number of live births,** median (IQR) | 2 (1.0) | 2 (1.0) | 2 (1.0) | 2.0 (1.0) | 0.27 |
| **Time since last pregnancy in years,** median (IQR) | 3.4 (7.4) | 1.9 (4.0) | 5.6 (8.1) | 15.6 (9.4) | 1.18 |

**^a^** Admission to intensive care unit, return to the OR, surgical complications, readmissions, and complications diagnosed during physician visits

^¶^SMD>0.1 means significant differences; **cannot publish <5 cell sizes because of privacy agreements with data stewards

Supplemental Table 9: Frequency of outcomes for people who received BS by age category in British Columbia between 2008-2022

|  | **< 35 years**  **(N = 3079)** | **35-45 (N = 3779)** | **> 45 years**  **(N = 244)** | **SMD** |
| --- | --- | --- | --- | --- |
| **Length of hospital stay in hours, median (IQR)** | 5 (1.0) | 5 (1.0) | 6 (2.0) | 0.07 |
| **OR time, median (IQR)** | 55 (17.0) | 56 (19.0) | 56 (18.0) | 0.04 |
| **Same day discharge (%)** | 3057 (99.3) | 3747 (99.2) | 240 (98.4) | 0.06 |
| **Any complication (%)** | 91 ( 3.0) | 100 ( 2.6) | 6 ( 2.5) | 0.02 |
| **Major complication^b^** | 21 (0.7) | 26 (0.7) | <5 | 0.04 |
| **Minor^c^** | 71 (2.3) | 76 (2.0) | <5 | 0.06 |
| **Intensive care unit (%)** | <5 | <5 | <5 | 0.02 |
| **OR return (%)** | <5 | <5 | <5 | 0.02 |
| **In hospital complications (%)** | 7 ( 0.2) | 7 ( 0.2) | <5 | 0.06 |
| **Readmission (%)** | 36 ( 1.2) | 40 ( 1.1) | <5 | 0.01 |
| **Readmission likely due to surgical complication (%)** | 13 ( 0.4) | 19 ( 0.5) | 1 ( 0.4) | 0.01 |
| **Number of physician visit, mean (SD)** | 1.2 (3.2) | 1.1 (3.1) | 1.2 (3.0) | 0.01 |
| **Physician visit - infection (%)** | 53 ( 1.7) | 56 ( 1.5) | <5 | 0.05 |
| **Physician visit - other complication (%)** | 22 ( 0.7) | 23 ( 0.6) | <5 | 0.03 |
| **Diagnostic test (%)** | 346 (11.0) | 449 (11.5) | 52 (13.8) | 0.06 |
| **X ray (%)** | 91 ( 3.0) | 126 ( 3.3) | 15 ( 6.1) | 0.10 |
| **Ultrasound (%)** | 69 ( 2.2) | 95 ( 2.5) | 6 ( 2.5) | 0.01 |
| **Lab test (%)** | 302 ( 9.8) | 386 (10.2) | 22 ( 9.0) | 0.03 |
| **Antibiotics (%)** | 283 ( 9.2) | 364 ( 9.6) | 21 ( 8.6) | 0.02 |
| Number of antibiotics days dispensed among users, mean (SD | 9.4 (8.6) | 10.1 (9.5) | 10.4 (7.4) | 0.10 |
| **NSAID (%)** | 868 (28.2) | 1108 (29.3) | 67 (27.5) | 0.03 |
| Number of NSAIDS days dispensed among users, mean (SD) | 9.7 (6.2) | 10.2 (7.8) | 11.4 (12.6) | 0.12 |
| **Opioids (%)** | 1584 (51.4) | 1874 (49.6) | 140 (57.4) | 0.10 |
| Number of days Opioids dispensed, mean (SD) | 4.3 (4.4) | 4.6 (6.2) | 4.6 (6.9) | 0.04 |

**^a^** Admission to intensive care unit, return to the OR, surgical complications, readmissions, and complications diagnosed during physician visits

^b^Admission to intensive care unit, return to the OR, in hospital surgical complications, readmissions

c Complications diagnosed during physician visits

SMD>0.1 means significant differences; **cannot publish <5 cell sizes because of privacy agreements with data stewards

Supplemental Table 10 Unadjusted and adjusted risk ratio for the outcomes by indication of BS in British Columbia between 2008-2022, missing data imputed using hot deck imputation

|  | **Contraception**  **(N = 6970)** | | **Prophylactic**  **(N = 132)** | | |
| --- | --- | --- | --- | --- | --- |
|  | Events | **Reference** | Events | **Crude risk ratio (95% CI )** | **Adjusted risk ratio (95% CI)** |
| **Same day discharge** | 6918 | 1.00 | 126 | 0.96 (0.93 - 1.00) | 0.96 (0.94 - 1.00) |
| **Any complication^a^** | 191 | 1.00 | 6 | 1.66 (0.75 - 3.67) | 1.81 (0.78 - 4.20) |
| **Diagnostic test^b^** | 771 | 1.00 | 18 | 1.23 (0.80 - 1.90) | 1.25 (0.80 - 1.96) |
| **Prescription analgesic use: NSAIDS** | 2002 | 1.00 | 41 | 1.08 (0.84 - 1.40) | 1.14 (0.88 - 1.49) |
| **Prescription analgesic use: Opioids use** | 3532 | 1.00 | 66 | 0.99 (0.83 - 1.17) | 0.93 (0.78 - 1.11) |

Models were adjusted by age, income, health authority and number of previous gynecologic surgeries.

**^a^** Admission to intensive care unit, return to the OR, surgical complications, readmissions, and complications diagnosed during physician visits

^b^Diagnostic test includes Xray, ultrasound or blood test

Supplemental Table 11 Unadjusted and adjusted risk ratios for the outcomes by age group when BS was received in British Columbia between 2008-2022, missing data imputed using hot deck imputation

| **< 35 years**  **(N = 3147)** | | | **>= 35 and <= 45years**  **(N = 3910)** | | | **> 45 years**  **(N = 377)** | | |
| --- | --- | --- | --- | --- | --- | --- | --- | --- |
|  | Events | **Reference** | Events | **Crude odds ratio (95% CI)** | **Adjusted odds ratio (95% CI)** | Events | **Crude odds ratio (95% CI)** | **Adjusted odds ratio (95% CI)** |
| **Same day discharge** | 3057 | 1.00 | 3747 | 1.00 (0.99 - 1.00) | 1.00 (0.99 - 1.00) | 240 | 0.99 (0.97 – 1.01) | 1.00 (0.98 – 1.01) |
| **Any complication^a^** | 91 | 1.00 | 100 | 0.90 (0.68 - 1.18) | 0.88 (0.66 - 1.17) | 6 | 0.83 (0.32 - 1.76) | 0.76 (0.34 - 1.73) |
| **Diagnostic test^b^** | 336 | 1.00 | 425 | 1.03 (0.90 - 1.18) | 1.03 (0.90 - 1.18) | 28 | 1.05 (0.73 - 1.51) | 1.04 (0.72 - 1.50) |
| **Prescription analgesic use: NSAIDS** | 868 | 1.00 | 1108 | 1.04 (0.96 - 1.12) | 1.05 (0.98 - 1.13) | 67 | 0.97 (0.79 - 1.20) | 1.03 (0.83 - 1.27) |
| **Prescription analgesic use: Opioids use** | 1584 | 1.00 | 1874 | 1.14 (0.96 - 1.12) | 0.96 (0.91 - 1.00) | 140 | 0.97 (0.79 - 1.20) | 1.09 (0.97 - 1.22) |

Models were adjusted by year of surgery, income, health authority and number of previous gynecologic surgery.

**^a^** Admission to intensive care unit, return to the OR, surgical complications, readmissions, and complications diagnosed during physician visits

^b^Diagnostic test includes, Xray, ultrasound or blood test
